# Increased TIM-3^+^PD-1^+^ NK cells are associated with the disease activity and severity of systemic lupus erythematosus

**DOI:** 10.1007/s10238-021-00726-8

**Published:** 2021-06-08

**Authors:** Qing Luo, Yunyuan Kong, Biqi Fu, Xue Li, Qingshui Huang, Zikun Huang, Junming Li

**Affiliations:** 1 Department of Clinical Laboratory, The First Affiliated Hospital of Nanchang University, Nanchang, 330006 Jiangxi People’s Republic of China; 2 Outpatient Department, The First Affiliated Hospital of Nanchang University, Nanchang, 330006 Jiangxi People’s Republic of China; 3 Department of Rheumatology, The First Affiliated Hospital of Nanchang University, Nanchang, 330006 Jiangxi People’s Republic of China

**Keywords:** Systemic lupus erythematosus, Natural killer cells, Programmed cell death protein 1, T cell immunoglobulin mucin-3

## Abstract

It is well established that natural killer (NK) cells are dysregulated in systemic lupus erythematosus (SLE) patients. However, the functions of NK cells and the mechanisms regulated by them in SLE remain incompletely understood. Patients with SLE were recruited from The First Affiliated Hospital of Nanchang University, and their clinical characteristics and treatments were recorded. The expression levels of T cell immunoglobulin mucin-3 (TIM-3) and programmed cell death protein 1 (PD-1) on NK cells were examined using flow cytometry. The correlations between the increase in TIM-3^+^PD-1^+^ NK cells in the SLE patients and clinical traits, including inflammatory markers, auto-antibodies, disease activity and severity of SLE, were examined. The TIM-3^+^NK cells, PD-1^+^NK cells and TIM-3^+^PD-1^+^ NK cells were significantly increased in the SLE patients. The increase in TIM-3^+^PD-1^+^ NK cells in the patients with SLE was associated with erythrocyte sedimentation rate, C-reactive protein, anti-double stranded DNA, anti-ribosomal P, SLE disease activity index and clinical features. The frequency of TIM-3^+^PD-1^+^NK cells in SLE patients with a cardiovascular disease (CVD) was significantly lower than that in SLE patients without a CVD. Moreover, the increased TIM-3^+^PD-1^+^ NK cells were significantly decreased in SLE patients following treatment. The present study suggested that the increased TIM-3^+^PD-1^+^ NK cells were associated with the disease activity and severity of SLE and may play a role in SLE pathogenesis.

**Supplementary Information:**

The online version contains supplementary material available at 10.1007/s10238-021-00726-8.

## Introduction

Systemic lupus erythematosus (SLE) is a systemic autoimmune disease characterized by the presence of autoantibodies that bind with self-antigens to form immune complexes that are deposited in various organs in which they may cause damage to those organs or entire organ systems [1, 2]. At present, the pathogenesis of SLE remains unclear. However, studies have demonstrated the role of natural killer (NK) cells in the development of SLE over the last few decades, showing that SLE patients had decreased numbers of NK cells in the peripheral blood, decreased NK cell cytotoxicity function, impaired NK cell differentiation and altered cytokine production from NK cells, and these NK cell defects may be associated with regulating activation of autoreactive lymphocytes [3–9].

NK cells are a subset of mononuclear cells, distinguished from B and T lymphocytes by virtue of their large granular morphology and the fact that they are part of the innate immune system [10]. NK cells are traditionally defined as CD56^+^CD3^−^ cells. Several NK cell surface activating receptors and inhibitory receptors have been verified to regulate function of NK cells, which results in NK cells modulating both the innate and adaptive immune responses [11].

Immune checkpoint receptors expressed by immune cells can negatively control their activation, expansion and effector functions via inhibitory signals generated by interacting with their cognate ligands. Our previous studies showed that abnormal expression of T-cell immunoreceptor with immunoglobulin and ITIM domains (TIGIT) on NK cells plays an important role in the pathogenesis of SLE, which suggests that the TIGIT signaling pathway may serve as a potential therapeutic target for treating this disease [12]. T cell immunoglobulin mucin-3 (TIM-3), a member of the TIM family, was discovered in 2001 [13] and acts as an important negative regulator of T cell-induced immune responses by interacting with its ligand, Galectin-9 [14]. Increasing evidence reveals that TIM-3 on immune cells is implicated in the pathogenesis of SLE [15, 16]. Recently, programmed cell death protein 1 (PD-1) has gained significant attention; PD-1 transmits inhibitory signals by binding with its ligand programmed death ligand 1 (PD-L1) to suppress the immune response following activation and proliferation of PD-1-expressing cells and thus maintains the balance of immune tolerance [17]. Abnormal PD-1 expression and function on immune cells were reported in SLE [18–20]. Moreover, Granier et al. [21] indicated that the percentage of tumor-infiltrating CD8^+^ T cells co-expressing PD-1 and TIM-3 was correlated with a more aggressive phenotype and a larger tumor size at diagnosis. The results also showed that co-expression of PD-1 and TIM-3 above the median conferred a higher risk of relapse and a poorer 36-month overall survival.

Considering the immunosuppressive role of TIM-3 and PD-1 in SLE, the fact that co-expression of PD-1 and TIM-3 on cells is correlated with disease clinical outcomes and that the cells co-expressing these proteins were dysfunctional, it was hypothesized that the co-expression of these two markers on NK cells may be associated with SLE and its clinical severity. Thus, the aim of this study was to detect the expression of TIM-3 and PD-1 on peripheral blood NK cells in SLE patients and controls to reveal the immune pathogenesis of SLE. The results suggested that the increase in TIM-3^+^PD-1^+^ NK cells was associated with disease activity and severity in SLE and may serve as a negative feedback mechanism, preventing potential tissue damage caused by excessive autoimmune responses in patients with SLE.

## Patients and methods

### Patients

A total of 44 patients with SLE who were admitted to The First Affiliated Hospital of Nanchang University were recruited to represent the SLE group. All cases were diagnosed in line with the revised American College of Rheumatology criteria for SLE [22]. Among all the SLE patients, 9 patients were re-examined after receiving regular treatment with immunosuppressive drugs and corticosteroids. The SLE disease activity index (SLEDAI) was used to calculate disease activity [23]. SLE patients were classified into an inactive group (SLEDAI score < 9) or an active group (SLEDAI score ≥ 10) according to SLEDAI [24]. Chronic damage in the SLE cohort was evaluated using the SLICC/ACR damage index (SDI) [25]. In the same period, 34 healthy subjects who did not exhibit any other comorbidities and who were unrelated to the SLE patients were selected as the healthy controls (HC). The patient characteristics of the two group are shown in Table 1. The present study was approved by the Ethics Committee of The First Affiliated Hospital of Nanchang University (approval on. 2,014,003) and complied with the Helsinki Declaration. All participants provided signed informed consent prior to participation.

**Table 1:** Clinical characteristics of patients with SLE and HC

Categories	SLE (*n* = 44)	HC (*n* = 34)
Females, *n* (%)	41 (93.2)	32(94.1)
Age, mean (S.D.), years	39.8 ± 15.3	40.8 ± 10.0
SLEDAI score, mean (S.D.)	8.2 ± 4.1	
SDI, mean (S.D.)	0.91 ± 1.1	
ds-DNA, mean (S.D.)	404.1 ± 522.8	
Anti-Sm, *n* (%)	10 (22.7)	
Anti-RIB-P, *n* (%)	15 (34.1)	
Anti-nucleosome, *n* (%)	14 (31.8)	
Anti-SSA, *n* (%)	22 (50.0)	
Anti-SSB, *n* (%)	5 (11.4)	
Anti-PL, *n* (%) (19 patients)	3 (15.8)	
C3, mean (S.D.)	0.6 ± 0.3	
C4, mean (S.D.)	0.1 ± 0.1	
IgG, mean (S.D.)	16.5 ± 7.8	
ESR, mean (S.D.)	51.6 ± 40.1	
CRP, mean (S.D.)	9.9 ± 12.8	
Clinical features		
Fever, *n* (%)	12 (9.1)	
Cutaneous manifestations, *n* (%)	10 (45.5)	
Oral ulcer, *n* (%)	5 (11.4)	
Alopecia, *n* (%)	2 (4.5)	
Arthritis, *n* (%)	8 (18.2)	
Effusion, *n* (%)	5 (11.4)	
proteinuria, *n* (%)	18 (40.9)	
Hematuresis, *n* (%)	9 (20.5)	
Pyuria, *n* (%)	6 (13.6)	
Leucopenia	8 (18.2)	
Erythrocytopenia	21 (47.7)	
Thrombocytopenia	9 (20.5)	
Anemia	25 (56.8)	
NPSLE	2 (4.5)	

### Flow cytometry analysis

Peripheral blood mononuclear cells were isolated from the fresh blood samples of SLE patients and HC using Ficoll-Paque gradient (Sigma-Aldrich, Merck KGaA, Darmstadt, Germany). The molecular phenotypes of NK cells were detected immediately using flow cytometry analysis. The following monoclonal antibodies were used: ECD-conjugated anti-CD3, PC7-conjugated anti-CD56 (cat. no. A07748, cat. no. A21692, Beckman Coulter, Inc., Brea, California, USA), and PE-conjugated anti-TIM-3, FITC-conjugated anti-PD-1 (cat. no. 85-12-3109-42, cat. no. 85-11-9969-42, MIH clones; eBioscience, Thermo Fisher Scientific, Inc., San Diego, California, USA). The NK cells were identified as CD56^+^CD3^−^ populations. Cells incubated with PE-conjugated mouse Immunoglobulin G (IgG) or FITC-conjugated mouse IgG antibodies (cat. no. A07796, cat. no. A07795, Beckman Coulter, Inc., Brea, California, USA) were used as isotype controls. All the cell suspensions with antibodies were incubated for 30 min on ice. Data were acquired on a CYTOMICS FC 500 flow cytometer (Beckman Coulter, Inc.) and analyzed using the associated software program (CXP 2.0, Beckman Coulter, Inc.).

### Serum IgG, complement 3 (C3), C4, C-reactive protein (CRP), autoantibody, erythrocyte sedimentation rate (ESR), urine and blood routine measurements

The levels of serum C3, IgG, C4 and CRP were detected by nephelometry according to the manufacturer’s protocol (IMMUNE800, Beckman Coulter, Inc.). Immunoenzyme dot assays (cat. no. DL 1590‑6401‑3G, Euroimmun AG, Lubeck, Germany) were used to detect anti-Sjögren's-syndrome-related antigen A (anti-SSA), anti-Sjögren's-syndrome-related antigen B (anti-SSB), anti-Smith (anti-Sm), anti-ribosomal P (anti-RIB-P) and anti-nucleosome antibodies according to the manufacturer’s instructions. Anti-double-stranded DNA (anti-dsDNA) of IgG class in serum was measured using commercially available ELISA kits (cat. no. KX‑E‑DSD01096, Kexin, Shanghai, China). Anti-phospholipid autoantibodies (anti-PL) of the IgG/IgM class, including an anti-cardiolipin antibody and an anti-β_2_ glycoprotein antibody in serum, were measured using commercially available ELISA kits (cat. no. EA 1621-9601 G/M, cat. no. EA 1632-9601 G/M, Euroimmun AG, Lubeck, Germany). ESR, and routine blood and urine measurements were determined according to the instructions described by the manufacturer (automatic measuring instrument for eSr Xc-40B, Pu li Sheng, China. Sysmex Xe-2100 analyzer, Sysmex, Kobe, Japan. Urine chemical analyzer Mejor-700I, Mei Qiao, China).

### Statistical analysis

All analyses were performed using SPSS version 16.0 (SPSS Inc., Chicago, Illinois, USA) or GraphPad Prism version 5.0 (GraphPad Software Inc., San Diego, California, USA). Differences in PD-1 and TIM-3 expression were analyzed using a Student’s t-test or a nonparametric Mann–Whitney U test. The correlation analysis was performed using Pearson’s correlation analysis or nonparametric Spearman’s correlation analysis. For evaluation of changes with treatment in the group of 10 patients, paired t-tests were used. *P* < 0.05 was considered to indicate a statistically significant difference.

## Results

### Expression of TIM-3 and PD-1 on NK cells in patients with SLE is increased

**Fig. 1:**
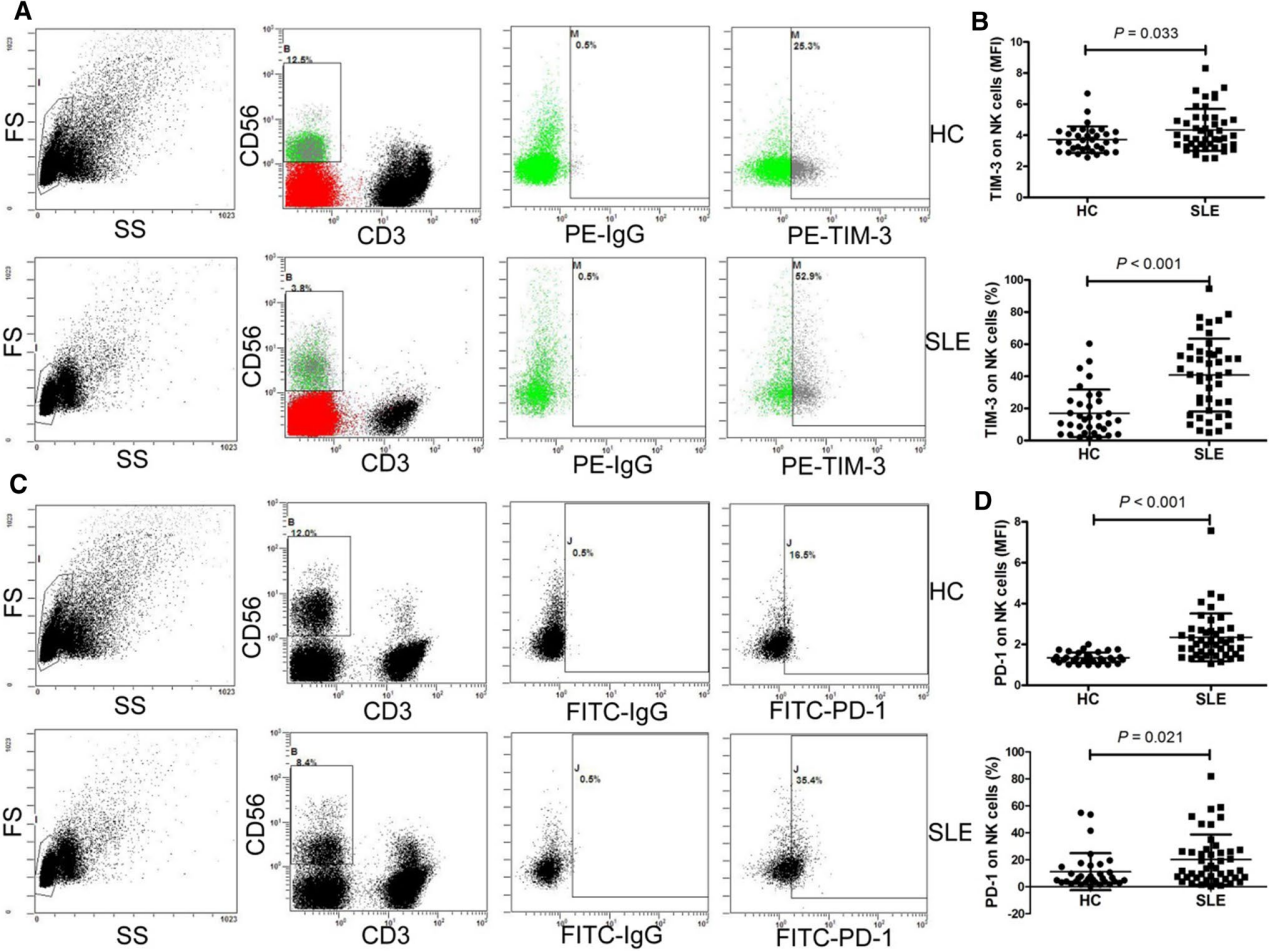
Expression of TIM-3 and PD-1 on NK cells in the SLE patients **a** Representative dot plots of population gating and TIM-3 expressing cells from the HC and SLE patients. **b** MFI of TIM-3 on NK cells and the frequency of TIM-3^+^NK cells were significantly increased in SLE patients. **c** Representative dot plots of population gating and PD-1 expressing cells from the HC and SLE patients. **d** MFI of PD-1 on NK cells and the frequency of PD-1^+^NK cells were significantly increased in SLE patients. HC, healthy control; MFI, mean fluorescence intensity; NK, natural killer; PD-1, programmed death 1; SLE, systemic lupus erythematosus; TIM-3, T cell immunoglobulin mucin-3

To determine the expression of TIM-3 and PD-1 on NK cells in SLE patients and the HC, their expression on NK cells was assessed using flow cytometry. We observed that the mean fluorescence intensity (MFI) of TIM-3 on NK cells, the frequency of TIM-3^+^ NK cells, the MFI of PD-1 on NK cells and the frequency of PD-1^+^ NK cells in SLE patients were all significantly increased compared with that in the HC (all *P* < 0.05) (Fig. 1).

### Increased TIM-3^+^PD-1^−^ NK cells and TIM-3^+^PD-1^+^ NK cells in patients with SLE

**Fig. 2:**
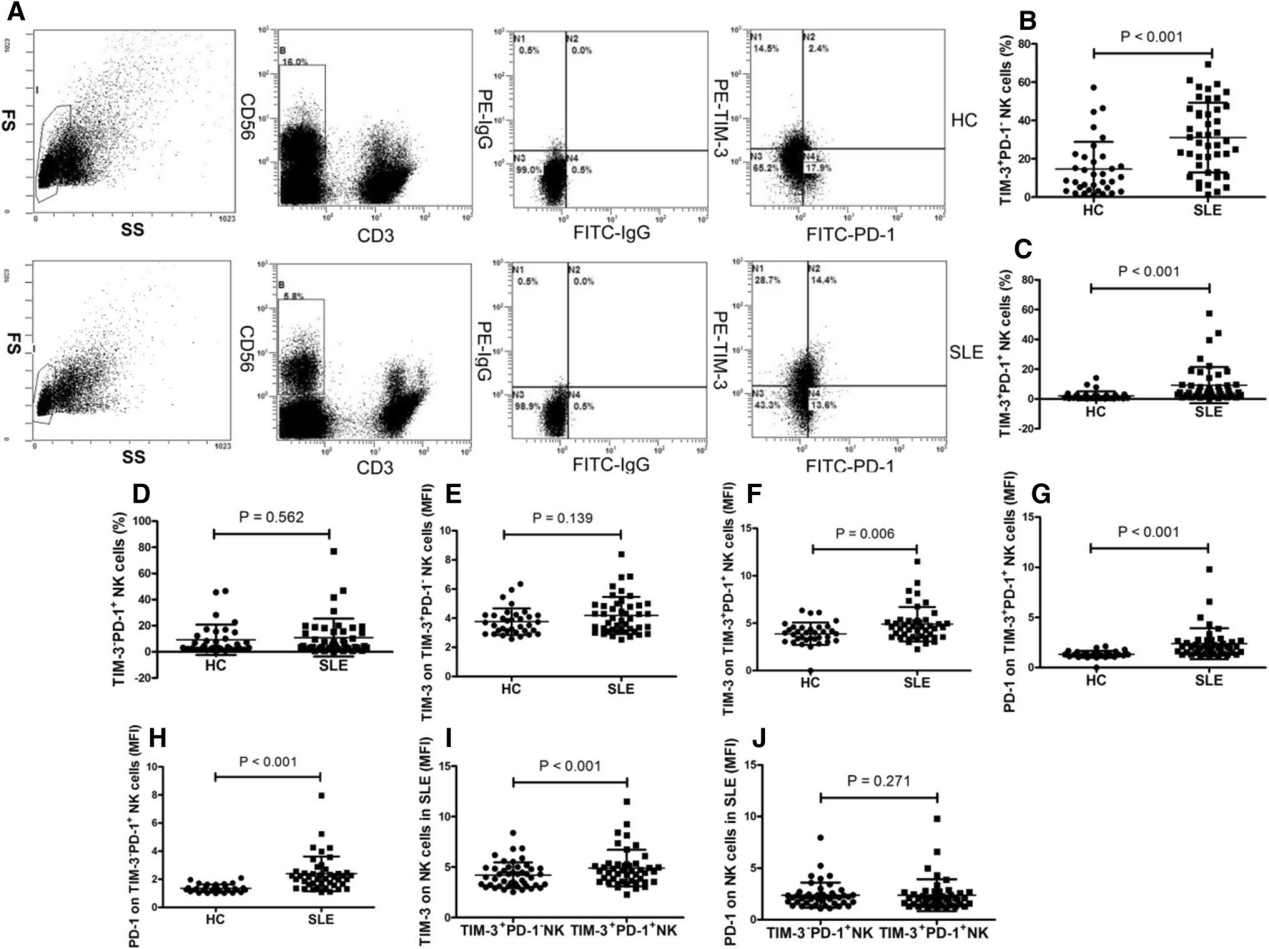
Expression of TIM-3 and PD-1 on TIM-3^+^PD-1^−^ NK cells, TIM-3^+^PD-1^+^ NK cells and TIM-3^−^PD-1^+^ NK cells in the SLE patients **a** Representative dot plots of population gating and TIM-3^+^PD-1 expressing cells from HC and SLE patients. **b** The frequency of TIM-3^+^PD-1^−^ NK cells was significantly increased in SLE patients compared with the HC. **c** The frequency of TIM-3^+^PD-1^+^ NK cells was significantly increased in SLE patients. **d** The frequency of TIM-3^−^PD-1^+^ NK cells was similar in HC and SLE patients. **e** The MFI of TIM-3 on TIM-3^+^PD-1^−^ NK cell was similar in the HC and the SLE patients. **f** The MFI of TIM-3 on TIM-3^+^PD-1^+^ NK cells was significantly increased in the SLE patients. **g** The MFI of PD-1 on TIM-3^+^PD-1^+^ NK cells was significantly increased in the SLE patients. **h** The MFI of PD-1 on TIM-3^−^PD-1^+^ NK cells was significantly increased in the SLE patients. **i** The MFI of TIM-3 on TIM-3^+^PD-1^+^ NK cells was significantly increased compared with the MFI of TIM-3 in TIM-3^+^PD-1^−^ NK cells in the SLE patients. **j** The MFI of PD-1 was similar in TIM-3^+^PD-1^+^ and TIM-3^−^PD-1^+^ NK cells in the SLE patients. HC, healthy control; MFI, mean fluorescence intensity; NK, natural killer; PD-1, programmed death 1; SLE, systemic lupus erythematosus; TIM-3, T cell immunoglobulin mucin-3

NK cells can be divided into three groups (TIM-3^+^PD-1^−^, TIM-3^+^PD-1^+^ and TIM-3^−^PD-1^+^) based on the expression of TIM-3 and PD-1. The frequencies of TIM-3^+^PD-1^−^ and TIM-3^+^PD-1^+^NK cells in SLE patients were significantly increased compared with that in the HC (both *P* < 0.05) (Fig. 2b, c), whereas the frequency of TIM-3^−^PD-1^+^ NK cells did not show any significant differences between the SLE patients and HC (*P* > 0.05) (Fig. 2d). The MFI of TIM-3 on TIM-3^+^PD-1^+^NK cells in SLE patients was significantly increased compared with that in the HC (*P* < 0.05) (Fig. 2f), whereas the MFI of TIM-3 on TIM-3^+^PD-1^−^ NK cells did not differ significantly between the SLE patients and the HC (*P* > 0.05) (Fig. 2e). The MFI of PD-1 on TIM-3^+^PD-1^+^ and TIM-3^−^PD-1^+^NK cells was significantly elevated in the SLE patients compared with the HC (*P* < 0.05) (Fig. 2g, h). In addition, the MFI of TIM-3 on TIM-3^+^PD-1^+^ NK cells was significantly elevated compared with that in the TIM-3^+^PD-1^−^NK cells in the SLE patients (*P* < 0.05) (Fig. 2i); however, the MFI of PD-1 on the TIM-3^+^PD-1^+^ NK cells and TIM-3^−^PD-1^+^ NK cells did not show any notable differences (*P* < 0.05) (Fig. 2j). These results showed that the proportion of TIM-3^+^PD-1^+^ NK cells in SLE patients was higher than that in the HC.

Next, we compared the percentage of NK cells, NK cell counts and TIM3^+^PD1^+^NK cell counts between SLE patients and HC. The percentage of NK cells and NK cell counts in the SLE patients was significantly lower than that in the HC (Fig. S1a, b), and there was a trend toward elevated TIM3^+^PD1^+^NK cells count in the SLE patients, although the difference was not significant (Fig. S1c).

### Increased TIM-3^+^PD-1^+^ NK cell levels are correlated with markers of inflammation

**Fig. 3:**
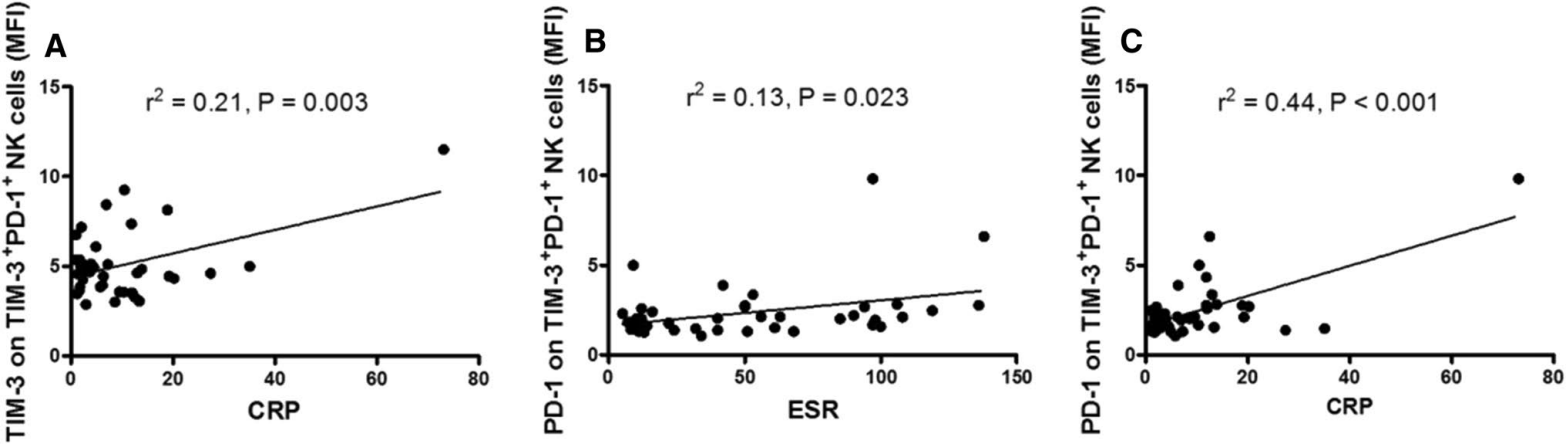
Increased levels of TIM-3^+^PD-1^+^ NK cells are correlated with ESR and CRP in the SLE patients **a** The MFI of TIM-3 on TIM-3^+^PD-1^+^ NK cells was positively associated with CRP. **b** The MFI of PD-1 on TIM-3^+^PD-1^+^ NK cells was positively associated with ESR. **c** The MFI of PD-1 on TIM-3^+^PD-1^+^ NK cells was positively associated with CRP. CRP, C-reactive protein; ESR, erythrocyte sedimentation rate; MFI, mean fluorescence intensity; NK, natural killer; PD-1, programmed death 1; SLE, systemic lupus erythematosus; TIM-3, T cell immunoglobulin mucin-3

CRP, immunoglobulin, complement, neutrophil count, neutrophil percentage and ESR are common markers of inflammation, and these markers were detected in this study. The relationship between these markers and increased TIM-3^+^PD-1^+^ NK cell levels, including the frequency of TIM-3^+^PD-1^+^NK cells, the MFI of TIM-3 on TIM-3^+^PD-1^+^ NK cells and the MFI of PD-1 on TIM-3^+^PD-1^+^ NK cells, was analyzed. As shown in Fig. 3, the MFI of TIM-3 on TIM-3^+^PD-1^+^ NK cells was correlated with CRP (*r*^2^ = 0.21, *P* = 0.003), and the MFI of PD-1 on TIM-3^+^PD-1^+^ NK cells was correlated with ESR (*r*^2^ = 0.13, *P* = 0.023) and CRP (*r*^2^ = 0.44, *P* < 0.001), whereas no correlation was found between the frequency of TIM-3^+^PD-1^+^NK cells and ESR or CRP. In addition, no correlations were found between increased TIM-3^+^PD-1^+^ NK cells and other markers of inflammation.

### Increased TIM-3^+^PD-1^+^NK cell levels are correlated with the presence of autoantibodies

**Fig. 4:**
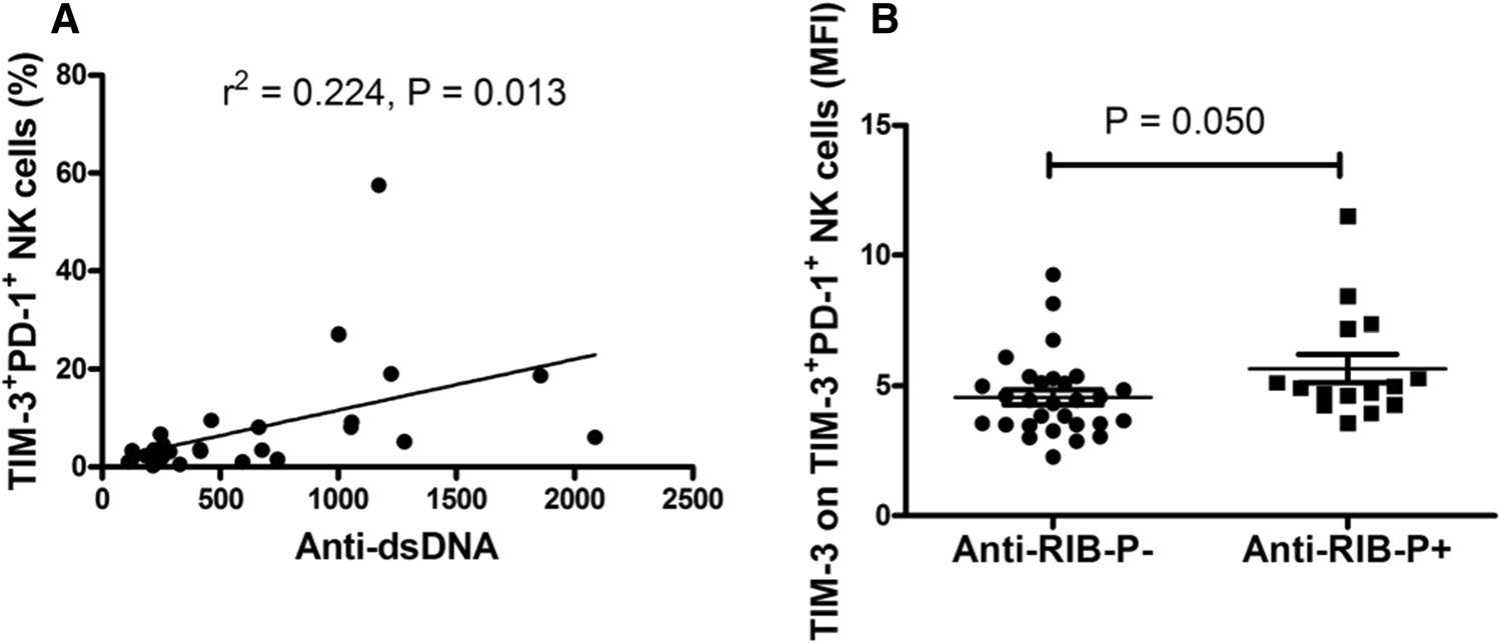
Increased TIM-3^+^PD-1^+^NK cells correlated with anti-dsDNA and anti-RIB-P in SLE patients **a** The frequency of TIM-3^+^PD-1^+^NK cells was positively associated with anti-dsDNA. **b** The MFI of TIM-3 on TIM-3^+^PD-1^+^ NK cells was significantly increased in anti-RIB-P positive SLE patients. Anti-dsDNA, anti-double-stranded DNA antibody; anti-RIB-P, anti-ribosomal P; MFI, mean fluorescence intensity; NK, natural killer; PD-1, programmed death 1; SLE, systemic lupus erythematosus; TIM-3, T cell immunoglobulin mucin-3

Anti-dsDNA, anti-nucleosome, anti-RIB-P, anti-SSA, anti-SSB and anti-Sm are hallmarks of autoantibodies in SLE, and anti-PL can also be detected in SLE patients. We explored the relationship between these autoantibodies and the increase in TIM-3^+^PD-1^+^NK cell levels, including the frequency of TIM-3^+^PD-1^+^NK cells, the MFI of TIM-3 on TIM-3^+^PD-1^+^NK cells and the MFI of PD-1 on TIM-3^+^PD-1^+^NK cells. As shown in Fig. 4, the frequency of TIM-3^+^PD-1^+^NK cells was positively correlated with anti-dsDNA (r^2^ = 0.22, P = 0.013), and the MFI of TIM-3 on TIM-3^+^PD-1^+^ NK cells was significantly elevated in anti-RIB-P positive SLE patients (*P* = 0.05). However, no correlation was found between increased TIM-3^+^PD-1^+^ NK cells and the presence of other autoantibodies.

### Increased TIM-3^+^PD-1^+^NK cell levels are correlated with disease activity and severity of SLE

**Fig. 5:**
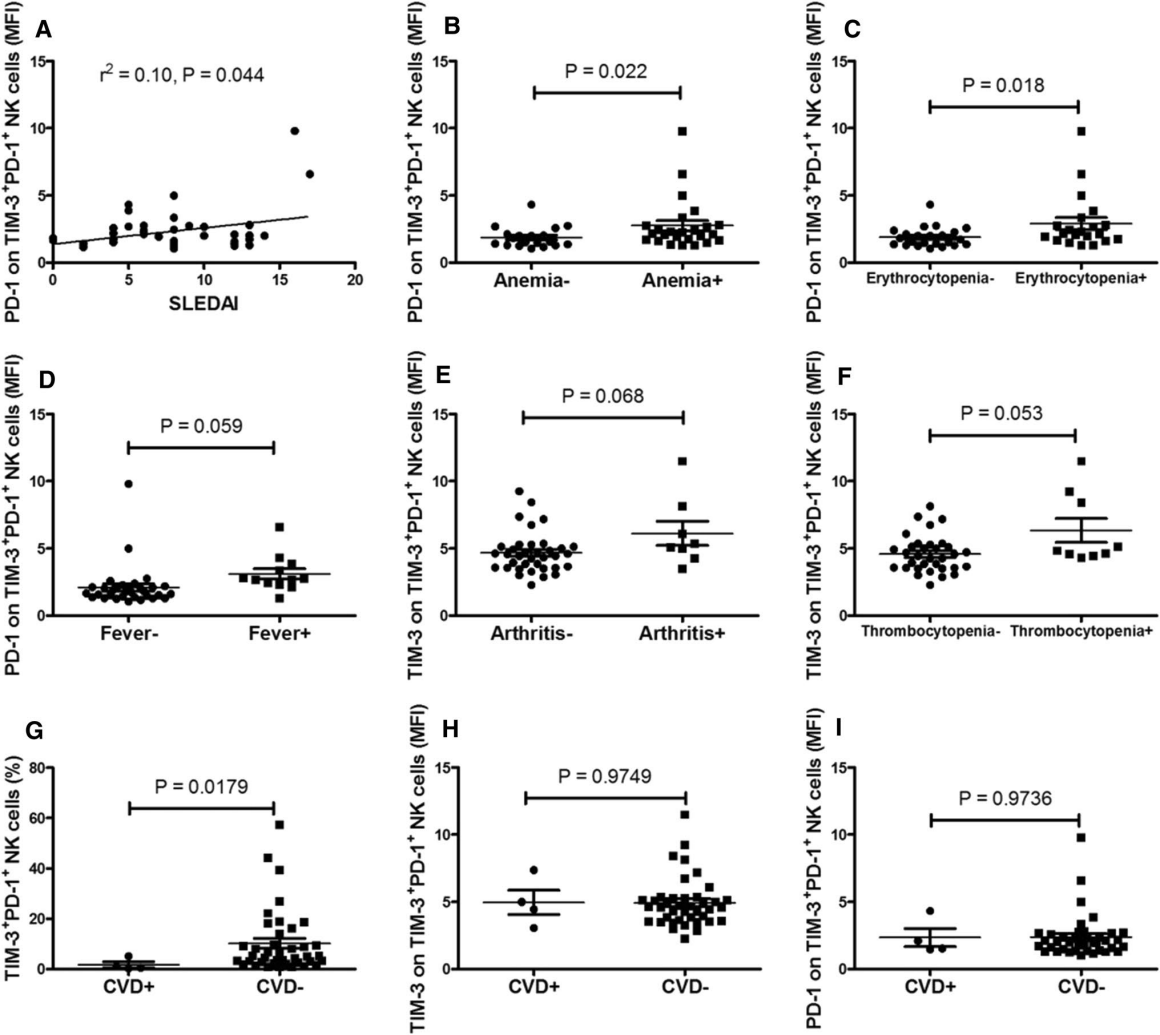
Increased levels of TIM-3^+^PD-1^+^NK cells are correlated with disease activity in the SLE patients **a** The MFI PD-1 on TIM-3^+^PD-1^+^ NK cells was positively correlated with SLEDAI score. **b** The MFI of PD-1 on TIM-3^+^PD-1^+^ NK cells was significantly increased in the SLE patients with anemia. **c** The MFI of PD-1 on TIM-3^+^PD-1^+^ NK cells was significantly increased in the SLE patients with erythrocytopenia. **d** The MFI of PD-1 on TIM-3^+^PD-1^+^ NK cells tended to be increased in the SLE patients with fever, but the difference was not significant. **e** The MFI of TIM-3 on TIM-3^+^PD-1^+^ NK cells tended to be increased in the SLE patients with arthritis, but the difference was not significant. **f** The MFI of TIM-3 on TIM-3^+^PD-1^+^ NK cells tended to be increased in the SLE patients with thrombocytopenia, but the difference was not significant. **g** The frequency of TIM-3^+^PD-1^+^NK cells in the SLE patients with CVD was significantly decreased compared with that in the SLE patients without CVD. **h** No correlation was found between the MFI of TIM-3 on TIM-3^+^PD-1^+^ NK cells and CVD or **i** between the MFI of PD-1 on TIM-3^+^PD-1^+^ NK cells and CVD. CVD, cardiovascular disease; MFI, mean fluorescence intensity; NK, natural killer; PD-1, programmed death 1; SLE, systemic lupus erythematosus; SLEDAI, SLE disease activity index; TIM-3, T cell immunoglobulin mucin-3

We also observed that the MFI of PD-1 on TIM-3^+^PD-1^+^ NK cells correlated positively with the SLEDAI score (*r*^2^ = 0.10, *P* = 0.044) (Fig. 5a). Furthermore, the SDI and the clinical signs of SLE patients, including cutaneous manifestations, fever, oral ulcer, arthritis, neuropsychiatric lupus (NPLE), alopecia, effusion, proteinuria, leucopenia, hematuresis, pyuria, thrombocytopenia, erythrocytopenia and anemia, were analyzed and correlated with increased TIM-3^+^PD-1^+^NK cells. As shown in Fig. 5, the MFI of PD-1 on TIM-3^+^PD-1^+^NK cells was significantly increased in the SLE patients with anemia (*P* = 0.022) or erythrocytopenia (*P* = 0.018). In addition, the MFI of PD-1 on TIM-3^+^PD-1^+^ NK cells tended to be increased in SLE patients with fever (*P* = 0.059), and the MFI of TIM-3 on TIM-3^+^PD-1^+^ NK cells tended to be increased in SLE patients with arthritis (*P* = 0.068) and thrombocytopenia (*P* = 0.053), although these differences were not significant.

Cardiovascular disease (CVD) is a major complication of SLE and is now a leading cause of death in these patients. Thus, we investigated the relationship between CVD and increased TIM-3^+^PD-1^+^NK cell levels. As shown in Fig. 5g–i, the frequency of TIM-3^+^PD-1^+^NK cells in SLE patients with CVD was significantly decreased compared with the SLE patients without CVD (*P* = 0.0179), whereas no correlation was found between the MFI of TIM-3 and PD-1 on TIM-3^+^PD-1^+^ NK cells and CVD (*P* = 0.9749 and *P* = 0.9736, respectively).

**Fig. 6:**
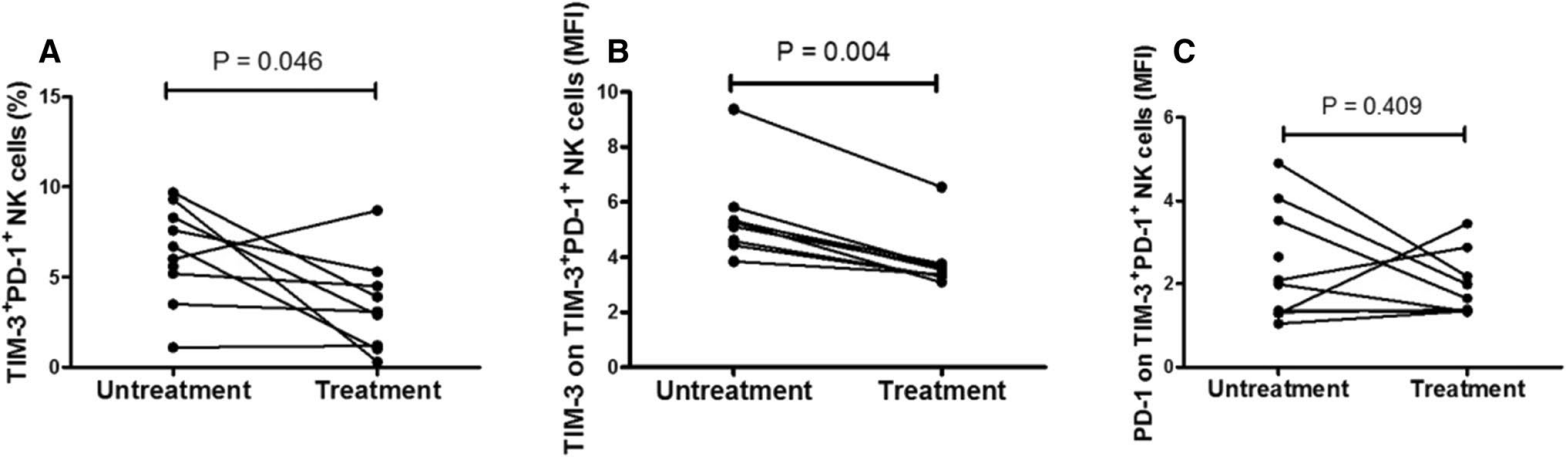
Increased levels of TIM-3^+^PD-1^+^ NK cells are correlated with treatment in the SLE patients **a** The frequency of TIM-3^+^PD-1^+^NK cells was significantly decreased in the SLE patients that received treatment. **b** The MFI of TIM-3 on TIM-3^+^PD-1^+^ NK cells was significantly decreased in the SLE patients that received treatment. **c** There was no difference in the MFI of PD-1 on TIM-3^+^PD-1^+^NK cells before and after treatment. MFI, mean fluorescence intensity; NK, natural killer; PD-1, programmed death 1; SLE, systemic lupus erythematosus; TIM-3, T cell immunoglobulin mucin-3

Moreover, we performed at least a 1-week follow-up estimate in 9 SLE patients who received treatment with 0.5–1.0 mg/kg/day corticosteroids or 0.5–1.0 mg/kg/day corticosteroids and immunosuppressive drugs (10–30 mg/kg/day mycophenolate mofetil or 3–5 mg/kg/day cyclosporine). As shown in Fig. 6, the frequency of TIM-3^+^PD-1^+^NK cells and the MFI of TIM-3 on TIM-3^+^PD-1^+^ NK cells were significantly reduced in the SLE patients who received treatment (*P* = 0.046 and *P* = 0.004, respectively), and no correlation was found between the MFI of PD-1 on TIM-3^+^PD-1^+^ NK cells with treatment.

## Discussion

NK cells are components of the innate immune system and are traditionally recognized as effector cells that directly kill infected or tumor cells. In recent years, there has been an increase in interest in interactions between NK cells and other immune cells, and studies have indicated that NK cells serve key roles in SLE [4, 26, 27]. However, little is known regarding the immunomodulatory effects of NK cells on the development of SLE. It is well known that the negative costimulatory molecules serve a key effect in regulating the activation conditions and functions of NK cells [12]. In this study, we explored the expression of Tim-3 on NK cells from SLE patients and found that its expression was significantly increased in SLE patients compared with HC, consistent with a previous report, which also showed that the expression of Tim-3 on CD56^+^ cells was increased in SLE patients [16]. To the best of our knowledge, the present study is the first to investigate the expression of PD-1 on NK cells from SLE patients and demonstrated that the expression of PD-1 on NK cells was significantly increased in SLE patients compared with the HC. The increased expression of PD-1 on NK cells was consistent with the results of PD-1 on T cells [18, 20].

Recently, increasing attention has been paid to the co-expression of PD-1 and TIM-3 on immune cells [28, 29]. The results of these reports indicated that co-expression of PD-1 and TIM-3 on T cells or NK cells was correlated with clinical outcomes, and the cells exhibited dysfunctional behaviors [21, 30]. In the present study, based on the expression of TIM-3 and PD-1, NK cells were divided into TIM-3^+^PD-1^−^ NK cells, TIM-3^+^PD-1^+^ NK cells and TIM-3^−^PD-1^+^ NK cells. The results showed that the frequency of TIM-3^+^PD-1^−^ and TIM-3^+^PD-1^+^NK cells was significantly elevated in SLE patients compared with the HC. Further analysis of the expression of PD-1 and TIM-3 on these NK cells found that the MFI of TIM-3 on TIM-3^+^PD-1^+^NK cells and the MFI of PD-1 on TIM-3^+^PD-1^+^ and TIM-3^−^PD-1^+^NK cells were significantly increased in SLE patients compared with the HC. These results indicated that the cell levels of TIM-3^+^PD-1^+^NK were increased, including the frequency of TIM-3^+^PD-1^+^NK cells, the MFI of TIM-3 on TIM-3^+^PD-1^+^NK cells and the MFI of PD-1 on TIM-3^+^PD-1^+^NK cells in SLE patients. Moreover, our research revealed that increased TIM-3^+^PD-1^+^ NK cell levels were associated with the SLEDAI score, clinical manifestation and treatment, which reflected disease activity and severity of SLE.

CVD is a major complication of SLE and is now a leading cause of death for these patients [31]. Moreover, invariant NK T cells may promote an atheroprotective effect in SLE patients with asymptomatic atherosclerotic plaques [32]. Thus, we investigated the relationship between CVD and increased TIM-3^+^PD-1^+^NK levels. The results showed that the frequency of TIM-3^+^PD-1^+^NK cells in SLE patients with CVD was significantly decreased compared with that in the SLE patients without CVD, suggesting that the increased frequency of TIM-3^+^PD-1^+^NK cells may exert an atheroprotective effect in SLE patients.

The frequency of TIM-3^+^PD-1^+^NK cells in SLE patients was significantly increased compared with that in HC, but the difference in TIM3^+^PD1^+^NK cell counts did not differ significantly. A number of studies have shown that the proportion of NK cells in SLE patients is decreased [12, 33]. In the present study, we also found that the proportion of NK cells in SLE patients was significantly decreased compared with the HC. It was suggested that the significantly decreased proportion of NK cells and NK cell counts in SLE patients resulted in the discrepancies between the frequency of TIM-3^+^PD-1^+^NK cells and TIM3^+^PD1^+^NK cell counts.

Autoimmune diseases, including SLE, are a type of chronic inflammation. Inflammation indices, such as ESR, CRP, C3 and C4, may mirror the severity of chronic inflammation [34]. Our results showed that the MFI of TIM-3 on TIM-3^+^PD-1^+^ NK cells was positively associated with CRP, and the MFI of PD-1 on TIM-3^+^PD-1^+^ NK cells was associated with ESR and CRP. These results suggested that increased TIM-3^+^PD-1^+^ NK cells could mirror the severity of chronic inflammation in SLE.

SLE is characterized by high-titers of serological autoantibodies, including anti-dsDNA, anti-nucleosome, anti-RIB-P, anti-SSA, anti-SSB and anti-Sm. In the present study, the levels of these autoantibodies were first detected, and their relationship with increased TIM-3^+^PD-1^+^ NK cells was investigated. The results indicated that the frequency of TIM-3^+^PD-1^+^ NK cells correlated positively with anti-dsDNA; the MFI of TIM-3 on TIM-3^+^PD-1^+^ NK cells was significantly increased in patients with positive anti-RIB-P, which suggested that the increased TIM-3^+^PD-1^+^ NK cells may be associated with the autoimmune response in SLE.

Several studies have reported the association between the positivity of anti-RIB-P and NPLE [35, 36]. In this study, only 2 SLE patients exhibited NPLE. In these 2 SLE patients with NPLE, only one was positive for anti-RIB-P and the positivity of anti-RIB-P was 50%. In the other 42 SLE patients without NPLE, 14 were positive, and the positivity of anti-RIB-P was 33.3%. Although the positivity of anti-RIB-P in SLE patients with NPLE exceeded the positivity of anti-RIB-P in SLE patients without NPLE, considering the low number of patients with SLE with NPLE as well, it was not statistically possible to investigate the correlation between the MFI of TIM-3 on TIM-3^+^PD-1^+^ NK cells and anti-RIB-P in SLE patients with NPLE. Thus, in future studies, larger sample sizes of SLE patients with NPLE are required to explore the correlation between the MFI of TIM-3 on TIM-3^+^PD-1^+^ NK cells and anti-RIB-P positivity in SLE patients with NPLE.

It is well established that PD-1 and TIM-3 are negative costimulatory molecules that are traditionally considered inhibitory molecules for the function of immune cells. However, some studies also found that the expression of PD-1 and TIM-3 on immune cells is positively associated with IFN-γ levels in SLE [18, 37]. In addition, other studies have shown that the expression of PD-1 and TIM-3 on T cells in SLE patients was correlated with autoantibody levels, such as that of anti-dsDNA [15, 20]. These studies suggest that PD-1 and TIM-3 not only serve an inhibitory role as a negative costimulatory molecule, but may also play a role in SLE pathogenesis by other means. Considering the effect of PD-1 and TIM-3, and the fact that the increased TIM-3^+^PD-1^+^ NK cells were associated with disease activity and severity of SLE, it is hypothesized that the increased TIM-3^+^PD-1^+^ NK cells may play a role in SLE pathogenesis via other mechanisms, such as serving as a negative feedback mechanism to prevent potential tissue damage caused by excessive autoimmune responses in patients with SLE.

## Conclusions

This study established a relationship between the increased TIM-3^+^PD-1^+^ NK cell levels with SLE disease activity and severity, and contributes to the understanding of the role of NK cells in SLE.

## Supplementary Information

Below is the link to the electronic supplementary material.

Figure S1. TIM3+PD1+NK cell counts, the frequency of NK cells and NK cell counts in the SLE patients and HC (a) There was no difference in TIM3+PD1+NK cell counts between HC and SLE patients. (b) The frequency of NK cells was significantly decreased in the SLE patients compared with the HC. (c) The NK cell counts was significantly decreased in the SLE patients compared with the HC. NK, natural killer; HC, healthy control; PD-1, programmed death 1; SLE, systemic lupus erythematosus; TIM-3, T cell immunoglobulin mucin-3. (DOCX 70 kb)

## Data Availability

All data generated or analyzed during this study are included in the published article.
