# Supplementary figures and images for: Increased TIM-3+PD-1+ NK cells are associated with the disease activity and severity of systemic lupus erythematosus
Source: Clin Exp Med. 2021 Jun 8;22(1):47–56. doi: 10.1007/s10238-021-00726-8 (PMC8863699; doi:10.1007/s10238-021-00726-8)

supplement Figure 1


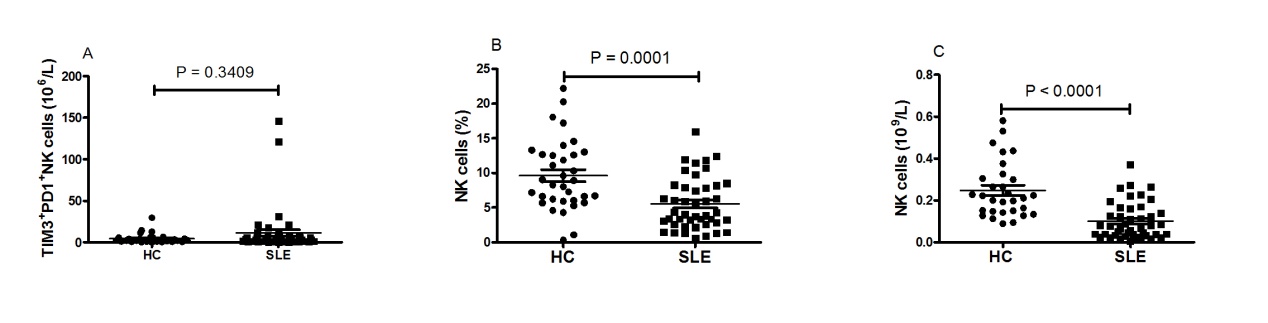

Supplement: Supplementary file 1 — Figure S1. TIM3+PD1+NK cell counts, the frequency of NK cells and NK cell counts in the SLE patients and HC (a) There was no difference in TIM3+PD1+NK cell counts between HC and SLE patients. (b) The frequency of NK cells was significantly decreased in the SLE patients compared with the HC. (c) The NK cell counts was significantly decreased in the SLE patients compared with the HC. NK, natural killer; HC, healthy control; PD-1, programmed death 1; SLE, systemic lupus erythematosus; TIM-3, T cell immunoglobulin mucin-3. (DOCX 70 kb) [file 10238_2021_726_MOESM1_ESM.docx]
